# Brighter Days Ahead for Suture-Mediated Closure: Safety, Selection, and Clinical Implications

**DOI:** 10.1016/j.jacadv.2025.102324

**Published:** 2025-11-13

**Authors:** Adriana C. Mares, Rahul Gupta, Bryan W. Kluck

**Affiliations:** a Georgetown University School of Medicine, Washington, DC, USA; b Yale New Haven School of Medicine, New Haven, Connecticut, USA; c Cardiovascular Experts of Pennsylvania, Camp Hill, Pennsylvania, USA

We congratulate Gaspardone et al^1^ for conducting the first-ever study consisting of the largest cohort and longest follow-up of patients undergoing a suture-mediated, deviceless approach to patent foramen ovale (PFO) closure. There were no findings of recurrent cerebrovascular events over 4 years—only 1 transient episode of atrial fibrillation (AF), which emphasizes the effectiveness and safety of this nondevice closure strategy. Unlike AF seen in device-associated PFO closure, risk of postprocedural AF was minimal in this study making it a valuable option for those prone to arrythmias and also for those with nickel sensitivity or allergy, provided anatomy is suitable (excluding fenestrated PFO’s).^2^^,^^3^ Successful closure rate at the follow-up was somewhat lower than traditional device-based closure (88.3% vs 93% to 100%). Residual left-to-right shunt after closure was predicted by wider PFO, septal aneurysm, and grade 3 or higher shunt on Valsalva. In this lengthy follow-up, the NobleStitch procedure was most often completed successfully with single and occasional need for additional sutures. The authors of this study were the first to report on the length, aneurysm, severe shunt, opening scoring system, a useful evaluative tool that could prove to be a good tool after broader standardization and external validation.

Interestingly, 10% of those with residual shunts were without recurrent neurologic or embolic events at the follow-up. This contrasts with prior device-based studies linking adverse neurological outcomes and residual shunt. This major difference between device and nondevice PFO closure strategies suggests that residual shunting is not necessarily the sole indicator of this feared complication.

This difference may reflect the impact of devices on atrial function, where altered physiology combined with residual shunt increases event risk, rather than the shunt alone.^1^ Moreover, many device studies relied on transient ischemic attack as an endpoint—a diagnosis that is clinically variable, often subjective and without an imaging correlate, and prone to misclassification—raising questions about whether residual shunting itself is the true driver of adverse events.^4^

Suture-mediated closure could be a useful addition to device-based PFO closure in carefully selected patients performed by experienced operators. Further studies should also aim to assess the generalizability of the procedure among other operators worldwide. The current study suggests that although consideration of suture-based closure in the majority of PFO closure patients is warranted, those patients who may require future left-sided interventions (appendage closure, ablations, valvular interventions, among others.), those with nickel allergy, and those at a higher risk of postprocedural arrhythmia would certainly benefit immediately.
